# In vitro experiments and network pharmacology-based investigation of the molecular mechanism of neferine in the treatment of gastric cancer

**DOI:** 10.1371/journal.pone.0318838

**Published:** 2025-03-26

**Authors:** Shicong Huang, Yi Nan, Guoqing Chen, Na Ning, Yuhua Du, Shuai Duan, Weiqiang Li, Ling Yuan

**Affiliations:** 1 Pharmacy College of Ningxia Medical University, Yinchuan, Ningxia, China; 2 Key Laboratory of Ningxia Ethnomedicine Modernization, Ministry of Education, Ningxia Medical University, Yinchuan, Ningxia, China; 3 Department of Chinese Medical Gastrointestinal, The Affiliated TCM Hospital of Ningxia Medical University, Wuzhong, China; Helwan University, EGYPT

## Abstract

**Background** Gastric cancer is the world’s leading tumor disease in terms of morbidity and mortality and is currently treated clinically with a comprehensive approach based on surgery. Studies have demonstrated the antitumor effects of neferine, but the anti-cancer mechanism for gastric cancer is not yet clear. **Methods** The Pubchem and Swiss TargetPrediction databases were searched to retrieve the targets of action of neferine. Meanwhile, relevant gene expression data were downloaded by means of the Gene Expression Omnibus(GEO) database to screen for differential genes and build a drug-disease network. The selected genes were analysed by bioinformatics analysis. Finally, gastric cancer treatment potential of neferine was determined through molecular docking. The molecular mechanism of neferine in the treatment of gastric cancer was verified by CCK8 assay, monoclonal assay, apoptotic and cycle assay, qRT-PCR and Western Blot. **Results** The results of network pharmacological analyses illustrate that the core genes are closely related to apoptosis, cell cycle, and cell proliferation. Through molecular docking, it was confirmed that neferine were closely related to key proteins. The results of in vitro experiments indicated that neferine could significantly inhibit the viability of gastric cancer cells, induce apoptosis of gastric cancer cells, and block the cell cycle of gastric cancer cells in the G0/G1 phase. **Conclusion** In summary, neferine inhibited the proliferation of gastric cancer cells through the CDK4/CDK6/CyclinD1 complex. This study provides a theoretical basis for the treatment of gastric cancer with neferine and an idea for the development of neferine for gastric cancer.

## Introduction

Gastric cancer(GC) is one of the most common types in the world, with the 5th and 4th highest incidences and mortality rates. In 2020, there were approximately 1,090,000 new cases of GC and 770,000 deaths worldwide. Morbidity and mortality rates are about 5.6% and 7.7%, respectively [1]. With 43.9% of new GC cases and 48.6% of GC deaths worldwide occurring in China-the largest developing country in the world. In 2020, GC had the third highest incidence and mortality rate in China [2]. The most important risk factors are diet, lifestyle, genetic susceptibility, family history, etc. [3]. Surgery is the major therapies for GC at present, and postoperative radiotherapy and chemotherapy are also important tools to assist GC treatment [4]. However, surgical treatment carries the risk of recurrence as well as the increase in drug resistance and side effects arising from frequent radiotherapy and chemotherapy [5], the search for a natural drug with anti-cancer effects has become one of the hottest research topics nowdays [6].

Lotus heart is the dried young leaves and embryonic roots of the mature seeds of lotus (*Nelumbo nucifera Gaertn.*) in the Nymphaeaceae family, which has the effects of calming the mind, relieving cough and phlegm, lowering blood lipids, being hemostatic and anti-inflammatory, being an antioxidant, being anti-AIDS and anti-thrombotic, protecting the liver, lungs, and kidneys, and protecting the central nervous system [7]. The neferine alkaloid (molecular formula C_38_H_44_N_2_O_6_) is a bisbenzylisoquinoline alkaloid extracted from lotus seeds [8]. Researchs have proved that neferine has anticancer, antioxidant [9], anti-inflammatory [10], antidepressant, anti-anxiety, anti-dementia, and other central pharmacological effects [11], as well as antithrombotic effects [12].

Natural compounds have an absolutely necessary role in performing a prophylactic or therapeutic effect on carcinoma [13]. Currently, researchers have demonstrated the effectiveness of certain plant chemicals against tumor cell lines, indicating their possible effect in complementary carcinoma treatment [14]. Toumi et al. found that the ethyl acetate fraction of thyme (*Thymelaea hirsuta L.*) extracts induced cell cycle arrest and apoptosis by studying the effect of the extract on colorectal cancer. The ethyl acetate fraction also inhibits cell invasion, in part by decreasing integrin α5 expression and FAK phosphorylation [15]. Yukio Fujiawara et al. found through their study that belladonna saponin A and naringin inducing CD169-positive macrophages in lymph nodes may boost anti-tumour immune responses [16]. Pereira MXG et al. found that pozzolanic acid produced antitumor effects on acute myeloid leukemia cells by decreasing cell viability in all cell lines, inducing cell death, increasing sub-G0/G1 accumulation, activating the cysteinase pathway, altering cell cycle distribution, and inhibiting the catalytic activity of two human DNA topoisomerases [17]. Roy A et al. found that leukotanin exerted anticancer effects by inactivating Akt/NF-k8, MMP-9, and VEGF [18]. Sapio L et al. found that trichothecenes exerted anticancer effects by inducing mesenchymal-epithelial cell transformation, inhibiting cell growth and migration, and enhancing the sensitivity of cancer cells to conventional antitumor drugs [19].

A network pharmacology study uses bioinformatics to analyze and construct the drug-target-disease network and predict the mechanism of action of the studied drug on the target disease. By using GC-MS, network pharmacology, and molecular docking, the mode of action of Magnolia officinalis essential oil in treating acute pancreatitis was explored by Li et al [20]. Xiao et al. explored the potential mechanisms for treating systemic lupus erythematosus by means of a network pharmacology study on tretinoin [21]. According to Huang et al., chrysophylla inducing apoptosis in breast cancer cells involves network pharmacology and molecular docking [22]. Multitargeted drug action involves a complex process, network pharmacology is an excellent tool for predicting the action of a single agent when we conduct studies of monomeric therapeutic disease targets. In order to better understand the therapeutic mechanisms produced by neferine for GC. We make use of the Swisstargetprediction database to predict the targets of neferine and screened GC differential genes in the GEO database, and the two were taken to intersect. Protein-protein Interaction (PPI) network analysis as well as Gene Ontology (GO), Kyoto Encyclopedia of Genes and Genomes (KEGG), and Gene Set Enrichment Analysis (GSEA) analysis were performed on the intersecting targets. Then clinical correlation and molecular docking analysis were performed. The above method provides insight into the mechanism of therapeutic action of neferine in GC. Moreover, the viability of neferine on gastric cancer cells was detected by the CCK8 assay, the ability of neferine to inhibit colony formation was verified by the monoclonal assay, neferine induced apoptosis in gastric cancer cells and blocked the cell cycle by flow cytometry, and neferine inhibited the proliferation of gastric cancer by inhibiting the expression of the cycling complexes CDK4/CDK6/CyclinD1, as detected by the qRT-PCR and Western Blot.

## Materials and methods

**Fig 1:**
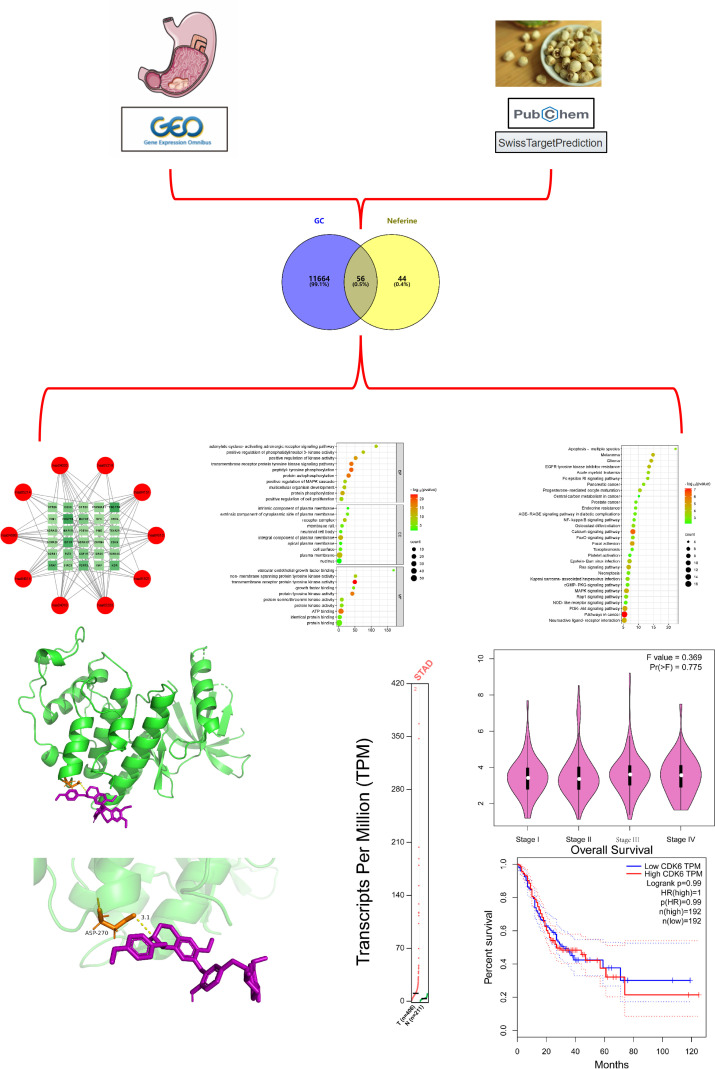
Flow diagram.

The steps described below are shown in Fig 1.

### Prediction of neferine target genes

The SMILES number of neferine was obtained from the Pubchem. The SMILES number was then used to acquire target genes in the SwissTargetPrediction.

### Gene microarray acquisition and differentially expressed gene screening for GC

The gene expression database GEO was cast about for the keyword “gastric cancer” to obtain the gene microarray data of GC, and then we downloaded the GSE49051 data set according to the study type “expression profiling by array” and the tissue source “homo sapiens.” We downloaded the GSE49051 dataset file and analyzed it by the GEO2R online tool that comes with the database and grouped the gene microarray data samples according to the disease The first three were in the normal group, the last three were in the GC group. Significantly different genes were selected according to the criteria of P. Value < 0.05 being significant and | logFC|>1. The significantly different genes with logFC > 1 were known as up-regulated genes, and those with logFC < -1 were known as down-regulated genes. The predicted targets of neferine and up- and down-regulated genes were plotted separately in Venn diagrams using the online platform Microbiology Letter, and the up- and down-regulated intersection genes were sorted according to differential expression ploidy and visualized as histograms, and the intersecting differential genes were plotted as polyheat plots for visualization. The differentially expressed genes were plotted as volcano plots using GraphPad Prism 9, and the annotations of the genes in the top ten positions of each of the intersecting differential genes were added.

### Gene acquisition of the neferine target for GC treatment

Significantly different genes for GC and predicted target genes for neferine were intersected at Venny 2.1.0 to acquire predictive genes for neferine for GC and plotted as a Venn diagram.

### Protein-protein interaction network and key target acquisition

Those obtained predicted target genes of neferine and GC were guided into the Search tool for the retrival of interacting genes/proteins (STRING) website for multiple protein analysis, with the biological species set to “Homo sapiens” and the rest as default. The network diagram in TSV format was downloaded and added into the Cytoscape 3.9.1, and the core target genes were filtered according to the degree of nodes (Degree) using the plug-ins CytoNCA and cytoHubba, and a topological analysis of the protein interaction network was constructed.

### GO biofunctional and KEGG pathway enrichment analysis

Those intersecting genes were imported into The Database for Annotation, Visualization, and Integrated Discovery (DAVID) website. Choosing Identifier as “OFFICE_GENE_SYMBOL”, List Type as “ Gene List” and “Homo sapiens” as the species to obtain GO biofunction and KEGG pathways. The results of the enrichment analysis were used to obtain core Biological Processes (BP), Molecular Functions (MF), Cellular Compositions (CC) and related biological pathways based on the results. The results were visualized according to Fold Enrichment, P value, Count using the online analysis tool Microbiology Letter Platform using bubble plots, with larger bubbles indicating more enriched genes and redder bubbles indicating smaller Pvalue values. The top 10 pathways and corresponding genes of KEGG enrichment analysis were ranked using Cytoscape 3.9.1 to construct a relationship.

### GSEA enrichment analysis

Gene information from the dataset in GSE49051 was analyzed for GSEA enrichment using the online analysis tool Microbiology Letter Platform.

### Molecular docking

Download the 3D structure map of neferine through the Pubchem, and screen the macromolecule corresponding to a single protein and method values as reference conditions through the PDB website. Download the 3D structure corresponding to core target. The small molecule ligands and water molecules of the corresponding proteins were deleted using Pymol, imported into AutoDock Tools software for hydrogenation, and then molecular docking was performed between the processed protein molecules and neferine, and the results were visualized by Pymol. The molecular docking energy was visualized using the online website heatmap.

### Clinical relevance analysis

The six core targets of molecular docking were visually analyzed using the Gene Expression Profling Interative Analysis (GEPIA) database with “STAD” as the keyword. The core targets were analyzed by stage analysis, copy number analysis, survival analysis, GC and para-cancer correlation analysis.

### In vitro experimental validation

#### Cells and reagents.

The human gastric cancer cell lines HGC-27 (Cat. No. CL-0107) and AGS (Cat. No. CL-0022) were purchased from Wuhan Prosperity Life Sciences Co. Fetal bovine serum (Cat. No. 900-18) was purchased from Gemini; RPMI 1640 (Cat.No.C11875500BT) was purchased from Gibco; and DMEM/F12 (Cat.No.SH30023.01) was purchased from Hyclone. The whole protein extraction kit (Cat. No. KGP2100) was purchased from Jiangsu Kaiji Biological Co., and the BCA protein content detection kit (Cat. No. ZJ102) was purchased from Yase Biomedical Technology Co. Antibodies CDK4, CDK6, CyclinD1, RB1, and p-RB1 were purchased from Wuhan Three Eagles Co. Trizol reagent (Cat. No. 15596026) was purchased from Invitrogen, USA; PrimeScriptTMRT reagent kit with gDNA eraser was purchased from Japan TaKaRa; and SuperReal fluorescence quantitative premix reagent was purchased from Tiangen Biochemical Technology Co.

#### Cell viability assay.

According to the concentration gradient, neferine was added at 0–60 μM and then incubated at 5% CO_2_ and 37°C for 12, 24, and 48 h. The CCK8 reaction mixture was prepared in the ratio of 1:10, and 100 μL of CCK8 reaction mixture was added into 96-well plates at 100 μL/well. The OD value of each well was detected at 490 nm with the zymography after incubation for 1 h. The IC50 value of each concentration was calculated according to GraphPad Prism 9. The IC50 value for each concentration was calculated according to GraphPad Prism 9.

#### Monoclonal formation assay.

Add 500 gastric cancer cells per well in a 6-well plate. After incubated at 5% CO2 at 37 °C for 24 h, three concentrations of neferine were calculated according to 2.9.1 were added at low, medium, and high concentrations, respectively, as well as a control group that was not given neferine intervention. After drug intervention, the medium was replaced with drug-free medium, and the fluid was changed every 3 days. Until a cell group of more than 50 cells was formed, the cells were fixed with 4% paraformaldehyde for 30 min, stained with 2% crystal violet for 15 min, washed with PBS three times, dried, photographed, counted. Count results were statistically analysed using GraphPad Prism 9 software.

### Detection of apoptosis by flow cytometry

Cells were spread in 60-mm petri dishes and incubated at 5% CO2 at 37 °C until the cell fusion reached 70%. Cells were collected after neferine intervention was added at three concentrations according to the low dose group, medium dose group and high dose group. The reaction solution was added according to the FITC-V/PI kit, and the apoptosis rate was detected on a flow cytometer three times in each group, and the statistical significance was counted using GraphPad Prism 9 software.

### Cell cycle detection by flow cytometry

Cells were collected after the dosing intervention as described in 2.9.3, the reaction working solution was configured according to the cycle kit and added to the cells of each group, and the apoptosis rate was detected on a flow cytometer three times for each group, and its statistical significance was counted using GraphPad Prism 9 software.

### qRT-PCR

Based on the results of network pharmacological analysis, molecular docking, and clinical analysis, *SYK*, *XIAP*, *ABCB1*, *FLT3*, *MAPK8*, and *CDK6* were selected to explore the mechanism of action of neferine in inhibiting gastric cancer proliferation. The specific primer sequences are shown in Table 1. HGC-27 human gastric cancer cells were used in the experiment. Total RNA was extracted from neferine-intervened gastric cancer cells using a medium RNA extraction kit, and reverse transcription and fluorescence quantification were performed according to the instructions. The relative expression of target genes was determined by the 2-ΔΔCt method.

**Table 1:** qRT-PCR primer sequence.

Gene	Primer sequence
***SYK*-F**	GGAGGAAGGCACACCACTACAC
***SYK*-R**	GCTTCTTGAGGAGGCAGACCAG
***XIAP*-F**	GCCAGACTATGCTCACCTAACCC
***XIAP*-R**	TTCTGACCAGGCACGATCACAAG
***ABCB1*-F**	GGTGCTGCTTTCCTGCTGATC
***ABCB1*-R**	TTCAATGCTTGGAGATGCCTGTC
***FLT3*-F**	AAGCGATGTATCAGAATGTGGATGG
***FLT3*-R**	GAGCCTGCGGAGAGAGTAGC
***MAPK8*-F**	CTCCACCACCAAAGATCCCTGAC
***MAPK8*-R**	CATTGACAGACGACGATGATGATGG
***CDK6*-F**	CCGAAGTCTTGCTCCAGTCCAG
***CDK6*-R**	GGGAAGGGCAACATCTCTAGGC

### Western Blot for CDK6, CDK4, CyclinD1, RB1, and p-RB1 protein expression

HGC-27 human gastric cancer cells were used in the experiment. Cells from the neferine intervention and normal groups were collected, added to cell lysates, and assayed for protein content using the BCA protein assay kit. Protein blot was transferred using PVDF membrane, the approximate location of corresponding proteins on the whole membrane was clipped according to the antibody description. CDK6, CDK4, CyclinD1, RB1, and p-RB1 antibodies were incubated at 4°C overnight. It is then mixed with secondary antibody and slowly shaken for 2 hours. The luminescent solution was added and exposed on an exposure machine. The results were calculated using Image J software.

### Statistical analysis

All statistical results were statistically analyzed using GraphPad Prism 9 software, and differences between groups were compared using one-way ANOVA. *P* < 0.05 was considered statistically significant.

## Results

### Prediction of neferine target genes

The SMILES numbers of neferine were obtained from the Pubchem database as CN1CCC2 = CC(=C(C = C2C1CC3 = CC = C(C = C3)OC)OC4 = C(C = CC(=C4)CC5C6 = CC(=C(C = C6CCN5C)OC)OC)O)OC, and 100 predicted genes were obtained useing the SwissTargetPrediction.

### Gene microarray acquisition and differentially expressed gene screening for GC

**Fig 2:**
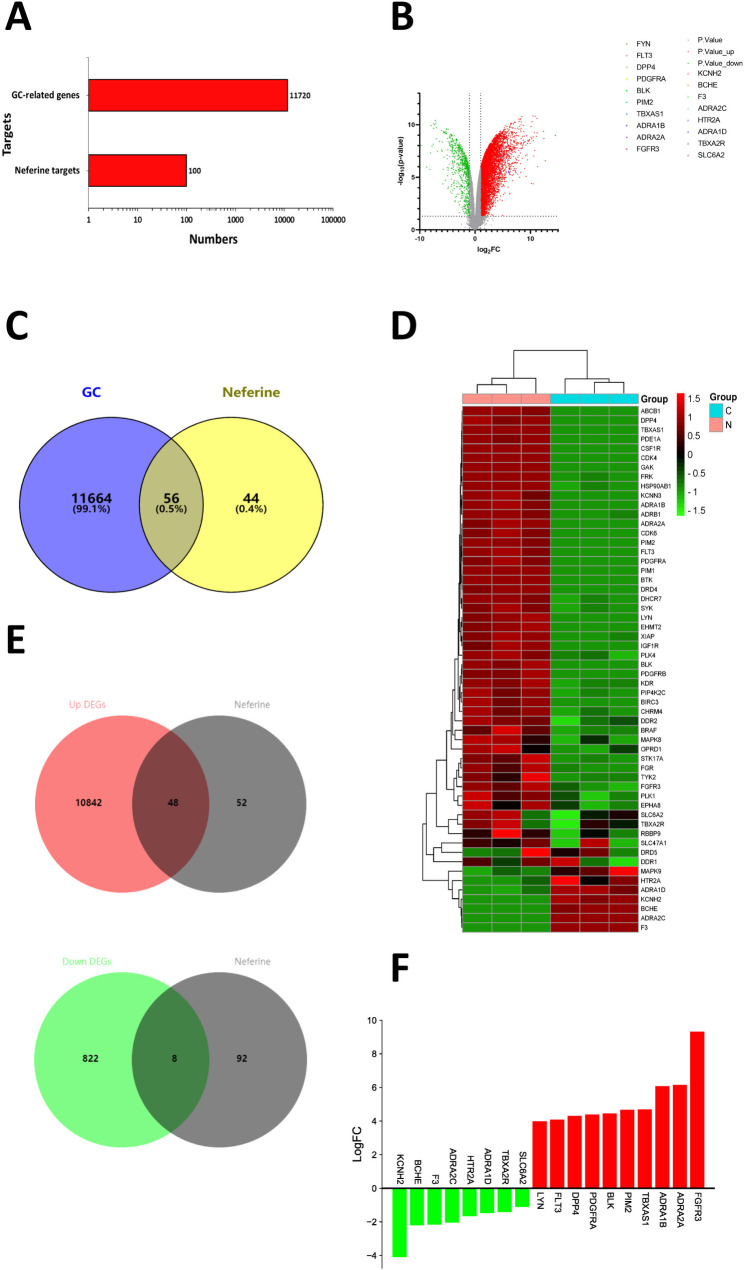
The intersection genes of neferine and GC and the down-regulated genes and the up-regulated genes on the intersection were visualized. **A:** The number of targets for neferine and GC; **B:** Differential gene volcano map; **C:** Venn diagram of the intersection of neferine and GC targets;**D:** Heat map of genes intersecting neferine and GC; **E:** Venn diagram of neferine and up- and down-regulated genes;**F:** Based on LogFC values, histogram comparisons were made between the top 8 down-regulated genes and the top 10 up-regulated genes.

We obtained 15962 GC gene microarray data by searching the gene expression database GEO with the keyword “gastric cancer” and then obtained 304 GC gene microarray data according to the study type “expression profiling by array.” The GSE49051 dataset file was selected to obtain normal gastric mucosal tissue samples and GC tissue samples. A total of 6 samples in this dataset, including 3 normal samples and 3 GC samples. The downloaded matrix file and the platform file were analyzed by the GEO2R online tool that comes with the database. And de-weighted to obtain a total of 22,684 genes in the dataset file, then 11,720 significant genes were obtained according to the criteria of P. Value < 0.05 being significant and | logFC|>1. The number of significantly different genes was compared with the predicted targets of neferine, as shown in Fig 2A. We have acquired 10,890 up-regulated genes and 830 down-regulated genes were screened according to the definition of up- and down-regulated genes. And intersected with the predicted targets of neferine to obtain 48 intersections of up-regulated genes and 8 intersections of down-regulated genes, as shown in Fig 2E. Those up-regulated gene intersections included *FGFR3*, *ADRA2A*, *ADRA1B*, *TBXAS1*, *PIM2*, etc. And the down-regulated gene intersections included *KCNH2*, *BCHE*, *F3*, *ADRA2C*, *HTR2A*, etc. Fig 2F shows the top 10 up-regulated and down-regulated genes. The genes in the data set were mapped as a volcano map, where the up-regulated genes were red, the down-regulated genes were green, and the non-differential genes were gray. We labeled the top 10 up-regulated genes and the top 8 down-regulated genes, as shown in Fig 2B.

### Gene acquisition of the neferine target for GC treatment

The 100 neferine predicted targets and 11,720 GC differential genes in the dataset were intersected at the Venn diagram website to make a Venn diagram, as shown in Fig 2C. A total of 56 intersecting targets were obtained, and the polyhedral map of the intersecting targets is shown in Fig 2D.

### Protein-protein interaction network and key target acquisition

**Fig 3:**
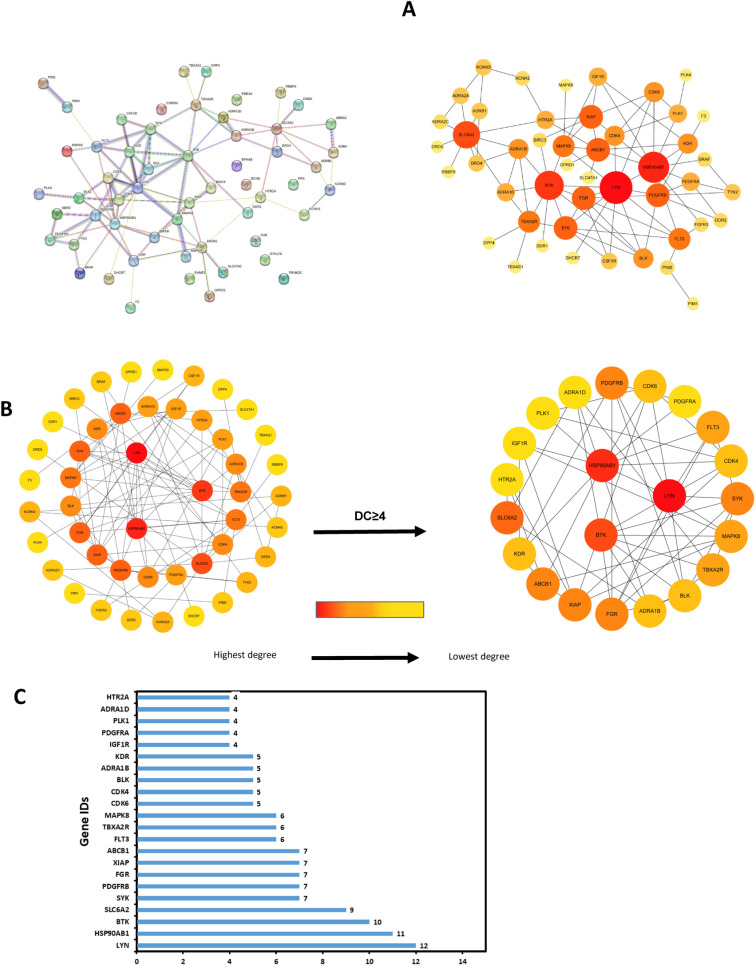
PPI enrichment analysis. **A:** PPI network diagram of the intersecting targets of neferine and GC;**B:** Screening of core target genes based on Degree value ≥  4; **C:** Degree values for core targets.

The intersecting targets were imported into the STRING website for multi-protein analysis, and the condition was set to “medium confidence (0.400)” to obtain 56 nodes and 92 relationship lines, and the free genes were removed, as shown in Fig 3A. The TSV format files were downloaded and imported into Cytoscape software, using CytoNCA and cytoHubba plug-ins, and the top 25 core target genes were filtered according to Degree ≥ 4 as shown in Fig 3B. Fig 3C shows the degree values of the top 25 core targets.

### GO biofunctional and KEGG pathway enrichment analysis

**Fig 4:**
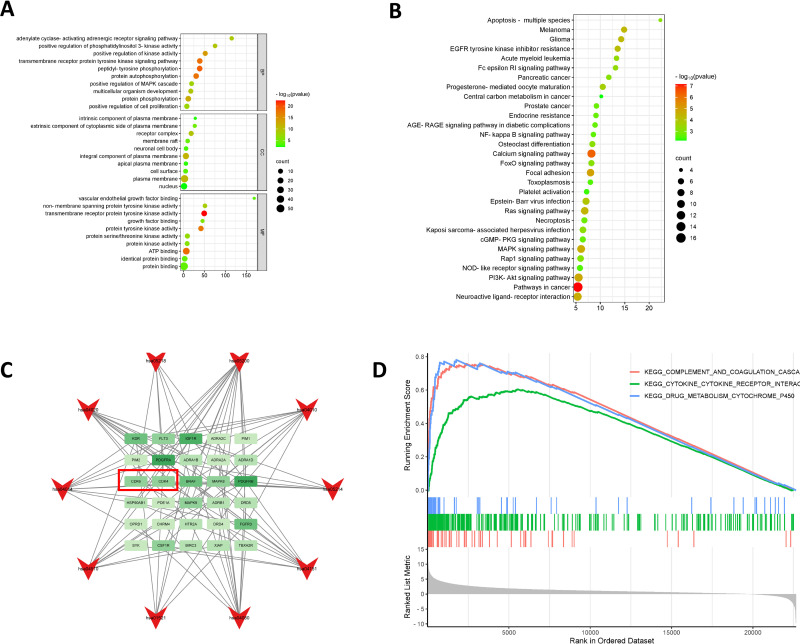
GO and KEGG Analysis. **A:** GO enrichment analysis; **B:** KEGG enrichment analysis;**C:** KEGG pathway and core target topology network diagram(In the red box are genes associated with the cell cycle);**D:** GSEA enrichment analysis.

The intersecting genes were entered into the DAVID database, and a total of 139 related biological processes, 43 molecular functions, 23 cellular compositions, and 58 related biological pathways were obtained. Fig 4A and 4B describe the top 10 related processes in the form of bubble diagrams of microbiological letter platforms according to fold enrichment, p value, and count. The top 10 related biological pathways and related genes were plotted in Cytoscape as a network diagram, as shown in Fig 4C.

### GSEA enrichment analysis

GSEA analysis of the genes in the dataset file in the microbiology letter platform shows that they are associated with complement and coagulation CASCA, cytokine interactions, and drug metabolism cytochrome P450, as shown in Fig 4D.

### Molecular docking

**Fig 5:**
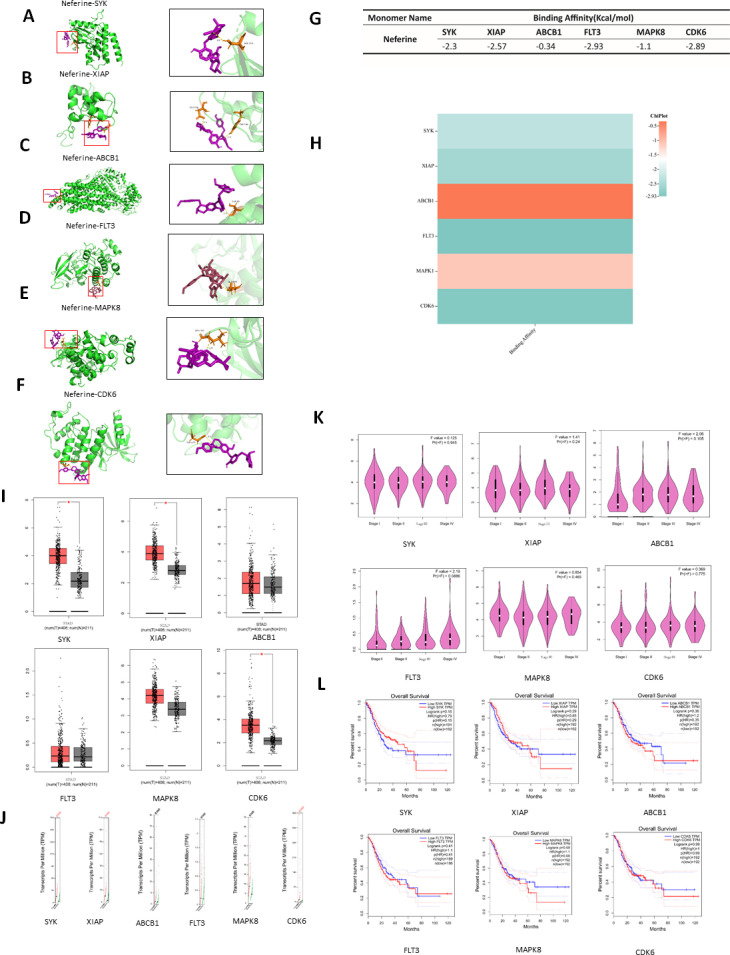
Clinical correlation analysis and molecular docking. **A:** Molecular docking visualization of Neferine-SYK; **B:** Molecular docking visualization of Neferine-XIAP; **C:** Molecular docking visualization of Neferine-ABCB1; **D:** Molecular docking visualization of Neferine-FLT3; **E:** Molecular docking visualization of Neferine-MAPK8; **F:** Molecular docking visualization of Neferine-CDK6; **G:** Molecular docking energy of the core target of neferine in the treatment of GC; **H:** Molecular docking energy heat map;**I:** Analysis of core gene GC and paracancer correlation;**J:** Graded staging analysis of GC with core genes; **K:** GC copy number analysis of core genes; **L:** Survival analysis of core genes in GC.

To test the possibility of neferine to remedy GC, the core targets were chosen for molecular docking in the order of DC value, from highest to lowest. Because some of the targets failed to dock with neferine, SYK, XIAP, ABCB1, FLT3, MAPK8, and CDK6 were chosen for molecular docking, and the results are shown in Fig 5A-F. The binding energy of each docked molecule was used to evaluate the possibility of successful docking; the docking energy of each protein docking result is shown in Fig 5G. According to the results, SYK, XIAP, FLT3, and CDK6 molecules have lower docking energies. The docking energy of each protein was visualized in a heat map of docking energy, and the results are shown in Fig 5H.

### Clinical relevance analysis

The *SYK*, *XIAP*, *ABCB1*, *FLT3*, *MAPK8*, and *CDK6* genes selected after comprehensive consideration were subjected to relevant clinical analysis in the GEPIA database, and *SYK*, *XIAP*, and *CDK6* were statistically significantly upregulated in the analysis of GC and paraneoplastic correlation, as shown in Fig 5I. In the analysis of graded staging and survival, *SYK*, *XIAP*, *ABCB1*, *FLT3*, *MAPK8*, and *CDK6* were not statistically significant, as shown in Fig 5J, L. In copy number analysis, *SYK*, *XIAP*, and *CDK6* were statistically significant, as shown in Fig 5K.

### In vitro experimental validation

#### Neferine inhibits cell viability.

**Fig 6:**
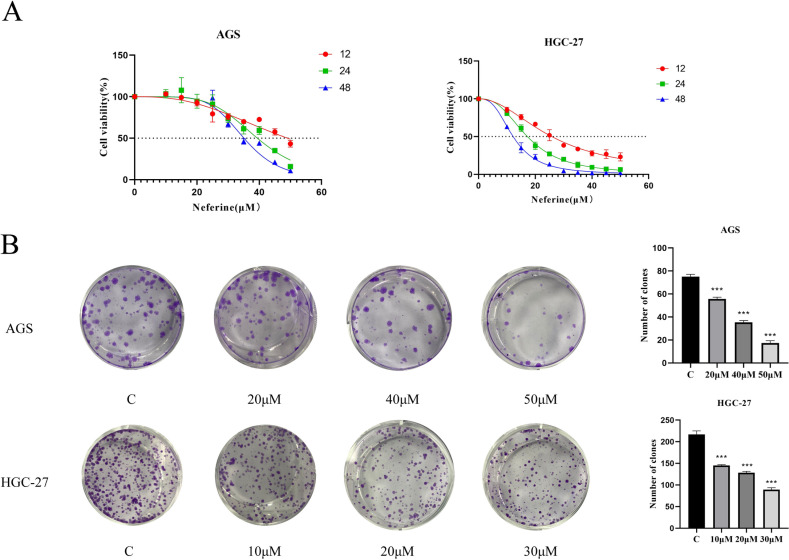
Cell viability was measured by CCK8 method and colony formation ability was verified by monoclonal assay. **A:** The cell viability of gastric cancer cells in AGS and HGC-27 was detected by CCK8 method; **B:** The ability of Neferine to interfere with the colony formation of AGS and HGC-27 gastric cancer cells was detected by monoclonal assay.(***P < 0.001).

To verify the inhibitory ability of neferine on the proliferation of gastric cancer cells, the viability of gastric cancer cells at each time period was determined using CCK8. With the increase in neferine administration concentration and time, the toxicity of neferine on AGS and HGC-27 gastric cancer cells was increasing gradually (see Fig 6A). According to the calculation results of GraphPad Prism software, the IC50 values of each group are shown in Table 2.

**Table 2:** Neferine intervention in AGS, HGC-27 cell IC50 values.

Cell type	12h	24h	48h
**HGC-27**	25.86μM	17.25μM	12.21μM
**AGS**	49.11μM	39.40μM	36.94μM

Based on the experimental results, HGC-27 and AGS cells intervention time of 24 h, The doses administered for specific follow-up experiments are shown in Table 3.

**Table 3:** Follow-up experimental dosage.

Cell type	Low dose	Medium dose	High dose
**HGC-27**	10μM	20μM	30μM
**AGS**	20μM	40μM	50μM

### Neferine inhibits cell colony forming ability

By monoclonal formation assay, it was found that according to the increase in neferine drug concentration, the inhibitory ability on cell colony formation also increased gradually and showed dose-dependence, as shown in Fig. 6B. It indicated that neferine could inhibit cell colony formation to suppress the proliferation of gastric cancer cells.

### Neferine induces apoptosis in gastric cancer cells

**Fig 7:**
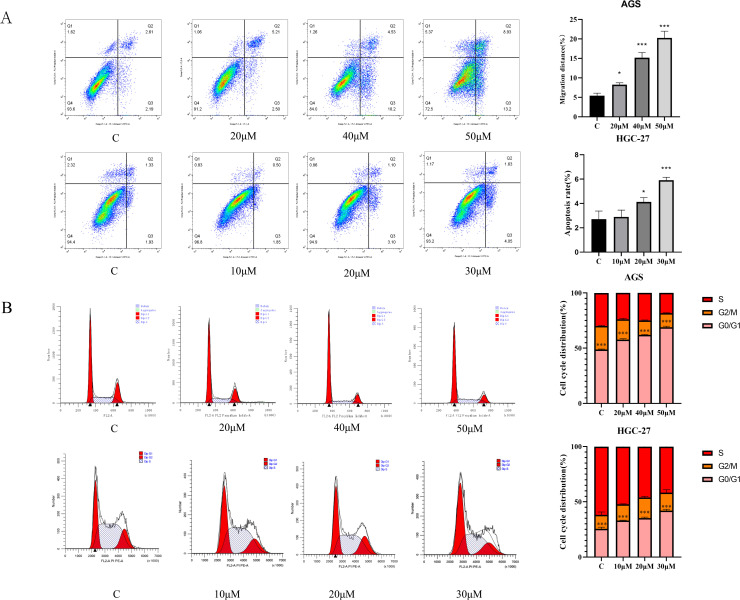
The apoptosis and cycle changes of gastric cancer cells were detected by flow cytometry. **A:** The effect of neferine on apoptosis rate of AGS and HGC-27 gastric cancer cells was detected by flow cytometry; **B:** The effect of neferine on the cell cycle of AGS and HGC-27 gastric cancer cells was detected by flow cytometry(***P < 0.001, **P < 0.01, * *P* < 0.05).

Apoptosis was detected using flow cytometry, and it was found that the apoptosis rate of gastric cancer cells showed an upward trend with the increase of neferine drug concentration in a dose-dependent manner, as shown in Fig 7A. It indicated that neferine could inhibit the proliferation of gastric cancer cells by increasing the apoptosis of gastric cancer cells. Although the apoptosis rate of the two kinds of cells was in line with the increasing trend, the change trend was not the same.

### Neferine blocks the cell cycle in gastric cancer

The cell cycle was detected using flow cytometry, and it was found that the change of the cell cycle of the two gastric cancers cells was more obvious. With the increase in neferine drug concentration, the gastric cancer cells were obviously blocked in the G0/G1 phase with a dose-dependent effect (see Fig 7B). It indicated that neferine could inhibit the proliferation of gastric cancer cells by blocking the gastric cancer cells in the G0/G1 phase.

### qRT-PCR

**Fig 8:**
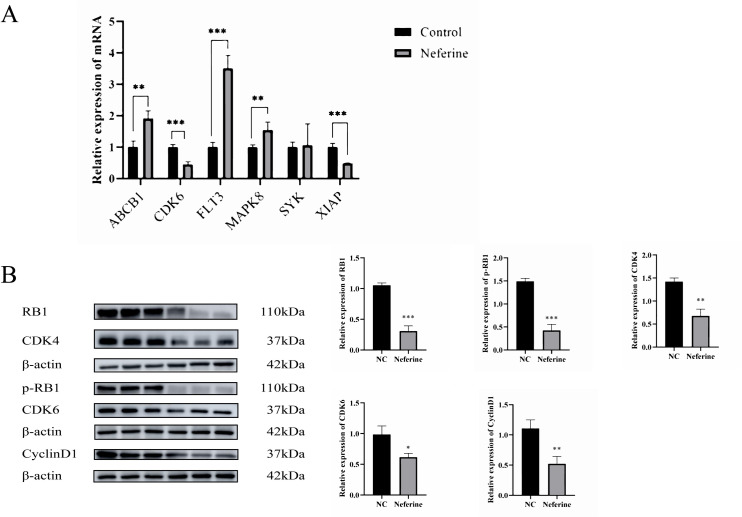
qRT-PCR was used to detect mRNA expression and Western Blot was used to detect cell cycle related proteins. **A:** The mRNA expression of core target was detected by qRT-PCR; **B**: Western Blot analysis was performed to detect the expression of cycle-related proteins after neferine intervention. Sections 1-3 are cut from the same blot, sections 4-6 are cut from the same blot, sections 7-8 are cut from the same blot. (***P < 0.001, **P < 0.01, * *P* < 0.05).

The six mRNAs selected for successful docking by neferine intervention 2.7 were detected using qRT-PCR, in which the mRNA expression of *ABCB1*, *FLT3*, and *MAPK8* showed an increase compared with the control group. mRNA expression of *CDK6* and *XIAP* showed a decrease compared with the control group. The mRNA of *SYK* showed no significant change compared with the control group, as shown in Fig 8A.

### Western blot for CDK6, CDK4, CyclinD1, RB1, and p-RB1 protein expression

Western Blot results showed that the protein expression of CDK4, CDK6, CyclinD1, RB1, and p-RB1 was decreased compared with that of the control group, and it can be concluded that neferine inhibits the phosphorylation of the downstream RB1 and thus inhibits the proliferation of gastric cancer cells by affecting the expression of CDK4/CDK6/CyclinD1 complex, as shown in Fig 8B.

## Discussion

In the current study, a total of 56 target genes and 58 related signaling pathways of neferine have a relationship with GC were mined by network pharmacology. PPI network analysis showed that neferine may regulate the anti-GC effect through *LYN*, *HSP90AB1*, *BTK*, *SLC6A2*, *SYK*, *PDGFRB*, and other targets. *SYK*, *XIAP*, *ABCB1*, *FLT3*, *MAPK8*, and *CDK6* genes that successfully docked with neferine were selected as the analysis objects. Among them, the docking of *FLT3* and *CDK6* with neferine was great combination of target protein and active compound. GO function and KEGG pathway enrichment results reveal the function, potential mechanisms, targets, and pathways that may be relevant for neferine treatment of GC, such as the PI3K-Akt pathway. Combined with the clinical correlation analysis, we hypothesized that neferine may affect the cell cycle of GC through *FLT3*, *CDK6*, and *SYK* to generate an anti-tumor effect.

Neferine was found to inhibit the cell viability of gastric cancer cells in vitro, induce apoptosis in a dose-dependent manner, block gastric cancer cells in the G0/G1 phase, and inhibit the ability of cell colony formation. By qRT-PCR, the core target of neferine was found to reduce the expression of *CDK6* mRNA and increase the expression of *ABCB1*, *MAPK8*, and *FLT3* mRNA and the Western blot showed that neferine significantly inhibited the expression of cycling-related proteins, such as CDK4, CDK6, CyclindD1, RB1, p-RB1, and so on. Therefore, we hypothesized that neferine could inhibit gastric cancer proliferation by suppressing the expression of the CDK4/CDK6/CyclinD1 complex.

Abnormalities in cell cycle progression are one of the basic mechanisms of tumorigenesis [23]. Cell cycle proteins control the phosphorylation of retinoblastoma protein and keep it in the G1 phase, which is one of the most common pathways of tumorigenesis [24]. CDK4 and CDK6 are key proteins of cellular entry into the S phase, which is critical for the onset and progression of many cancers [25]. A related study found that hepatogenic growth factor acts in conjunction with c-Jun to induce CCND1/CDK4/CDK6 expression and promote the proliferation of gliomas [26]. Neferine inhibits the proliferation of human gallbladder cancer cells through the CDK4/CDK6/CyclinD1 pathway [27]. Wang et al. found that tretinoin (TPL) induced bladder cancer cells to arrest in the G1 phase by inhibiting the expression of CDK4, CDK6, and CyclinD1 [28]. These studies suggest that the CDK4-CDK6-CyclinD1 pathway is associated with the abnormal cell cycle of cancer cells and is an important link with cancer development.

Currently, neferine are widely studied in cancers such as thyroid cancer [29], colorectal cancer [30], and prostate cancer [31]. Relevant studies have shown that neferine can activate the iron death-related signaling pathway Nrf2/HO-1/NQO1 to induce iron death in thyroid cells and inhibit their proliferation [29]; inhibit colon cancer cells by activating p65 and STAT3 [30] and they can activate the p38 and MAPK/JUK signaling pathways to induce the proliferation and migration of prostate cancer cells [30].Meanwhile, related studies have shown that neferine can also induce apoptosis in prostate cancer cells through autophagic flux and the JUK pathway [32]. Studies have shown that mitochondrial function is also one of the targets of neferine’s action, which is able to resist retinoblastoma proliferation by inducing its dysfunction [33]; neferine inhibits FAK and S6K1-expressed genes while inducing autophagy and apoptosis in retinoblasts [34]. Studies have shown that neferine can inhibit lung cancer cell growth by inhibiting the cell cycle and activating the MAPK signaling pathway [35]. At present, the mechanism of neferine in the treatment of gastric cancer has not been studied in depth.

In this experiment, the possible targets and signaling pathways of neferine for gastric cancer were predicted by network pharmacology, and the mechanism of action of neferine for gastric cancer was explored at the molecular level. The CCK8 assay was used to detect the viability of neferine on gastric cancer cells; the monoclonal assay was used to verify the ability of neferine to inhibit colony formation; flow cytometry was used to detect that neferine induced apoptosis and blocked the cell cycle of gastric cancer cells; and qRT-PCR and Western Blot were used to detect that neferine inhibited gastric cancer proliferation by inhibiting the expression of the cycling complexes CDK4/CDK6/CyclinD1. Next, we can carry out in vivo experiments on methylergonovine and analyze the metabolomics of drug-containing serum from experimental animals, so as to provide a more effective and reliable experimental basis for the treatment of gastric cancer with methylergonovine. Then the predicted main target genes were lentivirally transfected by the gene silencing method, and genomics, proteomics, and metabolomics analyses were carried out by the high-throughput screening method. Finally, the binding of transcription factors and related genes was detected by dual luciferase and chromatin immunoprecipitation methods, which provided new ideas for the study of neferine in the treatment of gastric cancer.

## Conclusion

Neferine inhibits the proliferation of gastric cancer cells by suppressing the expression of the cycling complex CDK4/CDK6/CyclinD1. Only in vitro experimental validation was performed in this manuscript, and in vivo experiments were not addressed. Subsequent in vivo experiments may be performed to validate the results.

## Supporting information

S2 Table 1 Gastric cancer genes.
Gastric cancer targets obtained from the GEO database.
(XLSX)

S2 Table 2 Neferine genes.
Drug targets obtained from the SwissTargetPrediction database.
(XLSX)

S2 Table 3 Intersection genes.
Intersecting genes of drugs and gastric cancer obtained by Venn diagrams.
(XLSX)

S2 Table 4 Heat map analysis.
Heatmap of the GEO database analysing the expression of intersecting genes across samples.
(XLSX)

S3 Table 1 DAVID analyse.
Results of GO and KEGG analyses of intersecting genes from the DAVID database.
(XLSX)

S4 Fig AGS, HGC-27 clone formation assay.
Pictures of the results of three experiments of AGS and HGC-27 clone formation experiments.
(TIF)

S8 Fig Results of Western blot experiments. Western blot uncut raw experimental results.
(TIF)
