# Supplementary figures and images for: In vitro experiments and network pharmacology-based investigation of the molecular mechanism of neferine in the treatment of gastric cancer
Source: PLoS One. 2025 Mar 26;20(3):e0318838. doi: 10.1371/journal.pone.0318838 (PMC11940423; doi:10.1371/journal.pone.0318838)

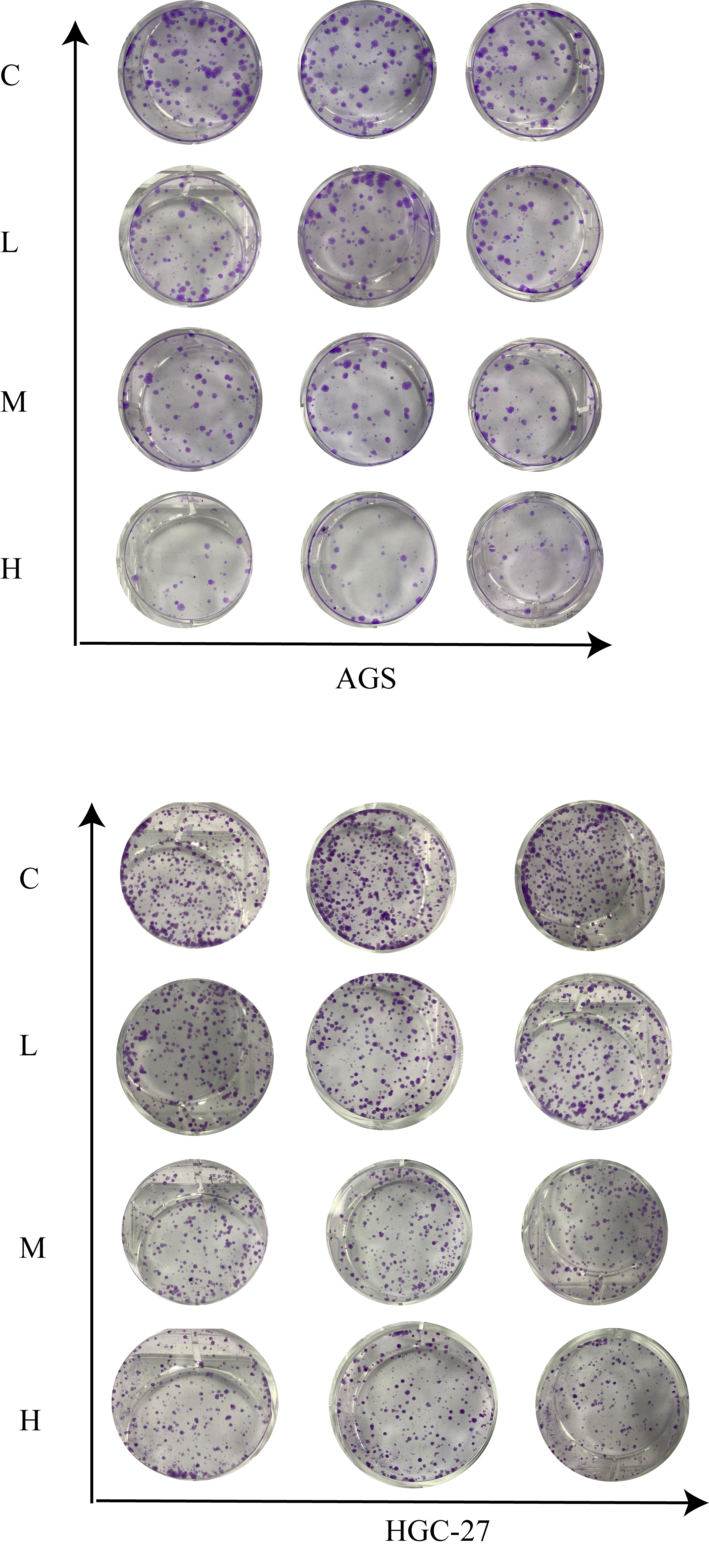

Supplement: S4 Fig — Pictures of the results of three experiments of AGS and HGC-27 clone formation experiments. (TIF) [file pone.0318838.s006.tif]

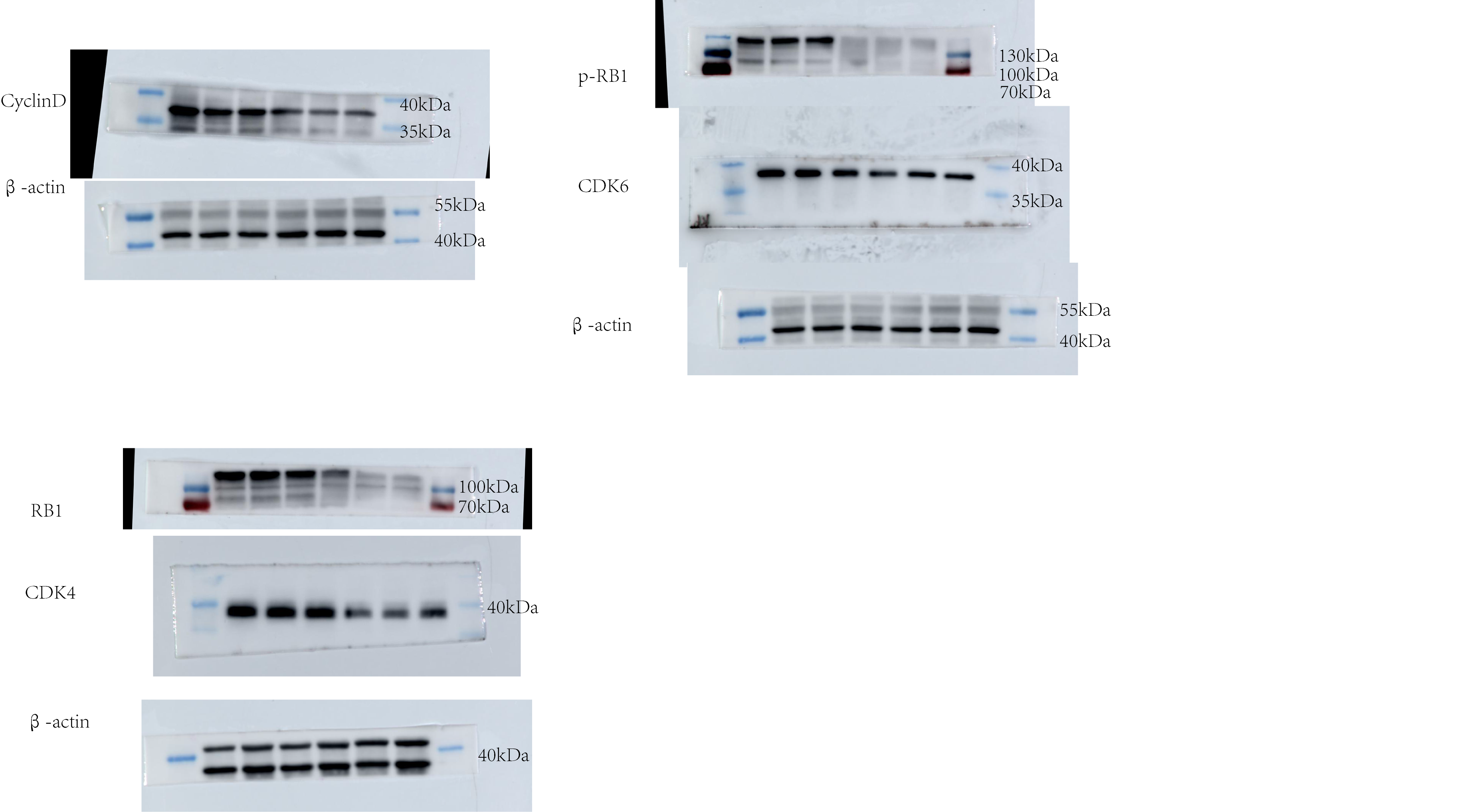

Supplement: S8 Fig — Western blot uncut raw experimental results. (TIF) [file pone.0318838.s007.tif]
